# Glucocorticoid receptor gene polymorphisms in hereditary angioedema with C1-inhibitor deficiency

**DOI:** 10.1186/s13023-016-0552-6

**Published:** 2017-01-10

**Authors:** Zsuzsanna Zotter, Zsolt Nagy, Attila Patócs, Dorottya Csuka, Nóra Veszeli, Kinga Viktória Kőhalmi, Henriette Farkas

**Affiliations:** 1 Hungarian Angioedema Center, 3rd Department of Internal Medicine, Semmelweis University, Kútvölgyi street 4, H-1125 Budapest, Hungary; 2 Department of Urology, Medical Center, Hungarian Defence Forces, Budapest, Hungary; 3 2nd Department of Internal Medicine, Semmelweis University, Budapest, Hungary; 4 HAS-SE “Lendület” Hereditary Endocrine Tumors Research Group, Hungarian Academy of Sciences, Budapest, Hungary

**Keywords:** Hereditary angioedema, C1-inhibitor deficiency, Trigger factor, Emotional stress, Coping, Glucocorticoid polymorphisms, Glucocorticoid sensitivity

## Abstract

**Background:**

Hereditary angioedema caused by C1-inhibitor deficiency (C1-INH-HAE) is a rare, autosomal dominant disorder. C1-INH-HAE is characterized by edema–formation, which may occur in response to stress. The individual’s response to stress stimuli is partly genetically determined. Activation of the hypothalamic–pituitary–adrenal axis results in the release of cortisol. In turn, the secreted gluco- and mineralocorticoids affect the metabolism, as well as the cardiovascular and immune systems. We hypothesized that changes in serum cortisol level and polymorphisms of the glucocorticoid receptor (GR) modify the individual sensitivity to stressor stimuli of C1-INH-HAE patients.

**Results:**

We compared the response to stress with Rahe’s Brief Stress and Coping Inventory of 43 C1-INH-HAE patients, 18 angioedema patients and 13 healthy controls. 139 C1-INH-HAE patients and 160 healthy controls were genotyped for glucocorticoid receptor polymorphisms BclI, N363S and A3669G. Serum cortisol levels were determined during attacks and during symptom-free periods in 36 C1-INH-HAE patients. The relationships between clinical, laboratory data and GR SNPs (Single Nucleotide Polymorphisms) were assessed using ANOVA. C1-INH-HAE patients have decreased coping capabilities compared to healthy controls. Cortisol levels were significantly higher during attacks than in symptom-free periods (*p* = 0.004). The magnitude of the elevation of cortisol levels did not show a significant correlation with any clinical or laboratory data. Among the C1-INH-HAE patients, the carriers of the A3669G allele had significantly lower cortisol levels, and increased body mass index compared with non-carriers.

**Conclusions:**

The higher cortisol level observed during attacks may reflect the effect of a stressful situation (such as of the attack itself), on the patients’ neuroendocrine system. In A3669G carriers, the lower cortisol levels might reflect altered feedback to the hypothalamic–pituitary–adrenal axis, due to decreased sensitivity to glucocorticoids.

## Background

Hereditary angioedema (HAE) with C1-inhibitor deficiency (C1-INH-HAE) is a rare, autosomal dominant disorder, which belongs to bradykinin-mediated angioedemas [1]. The deficiency of a serine protease protein C1-inhibitor (C1-INH) results in the activation of four plasma cascade systems (fibrinolytic, coagulation, kinin, and complement cascades), and this leads to the release of bradykinin from high-molecular-weight kininogen. Bradykinin, a vasoactive mediator, enhances capillary permeability. As a result, plasma leaks from the intravascular compartment into the extracellular space, leading to edema formation [2]. The episodes of angioedema may involve the subcutis and/or the submucosa in patients with HAE. Angioedema attacks may cause severe abdominal pain, which resembles that occurring in an abdominal emergency, or upper airway edema, which can lead to asphyxiation [3].

In general, C1-INH-HAE first occurs during the first decade of life [4, 5]. Although a consensus parameter defining the severity of HAE is lacking, it is characterized by the frequency of edematous attacks, subjective described attack severity and the need of on demand C1-INH substitution.

The factors, which may trigger an attack, include infections, emotional stress, physical exertion, trauma, invasive medical procedures, menstruation, and contraceptive use, as well as treatment with certain medications (i.e. ACE-inhibitors). In our recent study, we found that emotional stress is the most common trigger factor of attacks [6]. Chronic stress as a general risk factor for the development of several diseases; it can also modify disease activity [7–10].

Stressor stimuli activate the hypothalamic–pituitary–adrenal (HPA) axis, and result in the release of mineralo- and glucocorticoids (GCs). The sustained elevation of glucocorticoid (GC) levels has been associated with hypertension, weight gain, glucose intolerance, and hypertriglyceridaemia. GCs exert their diverse actions through the GC receptor (GR), which is ubiquitously expressed in many tissues and cell types [11]. Differences in individual glucocorticoid sensitivity may influence stress reactivity. Furthermore, altered glucocorticoid sensitivity has been shown to modify the manifestations of several diseases [12–14]. A few polymorphisms in the GR gene are known to modify glucocorticoid sensitivity. The BclI (rs41423247), a restriction fragment length polymorphism (RFLP), results from an intronic region (C/G) nucleotide substitution associated with increased glucocorticoid sensitivity, as well as with increased abdominal obesity, greater body mass index (BMI), decreased insulin sensitivity and dyslipidemia [15, 16]. The BclI polymorphism has been implicated in the pathogenesis or onset of various diseases [12–14, 17, 18]. In the central nervous system, it has been linked to mood disorders and to the responsiveness of the HPA axis [19, 20].

The N363S (rs6195) polymorphism in exon 2 of the *GR* gene, the (A/G) substitution causes an asparagine-to-serine change, associated with enhanced glucocorticoid sensitivity [21]. The results regarding the relationship of autoimmune diseases and carrier status are controversial [11]. This polymorphism has been described to modify disease symptoms patients with congenital adrenal hyperplasia (CAH), and may be involved in the pathogenesis of bilateral adrenal adenomas [22, 23]. The A3669G (GR-9ß, rs6198) polymorphism is located in the 3’ untranslated region of the *GR* gene. The (A/G) nucleotide substitution destabilizes the mRNA and causes a shift to the stabilization of the GRß (glucocorticoid receptor beta) splicing variant. The GRß isoform exerts a dominant, negative activity on the GRα (glucocorticoid receptor alpha) function, and the altered GRα/GRß ratio may lead to relative glucocorticoid resistance [24]. The A3669G polymorphism has been linked to a more active immune system [11], and to the development of rheumatoid arthritis [25]. The A3669G SNP was also attributed a role to bipolar diseases and depressive disorders [26, 27].

In this study, we investigated whether the clinical manifestations of C1-INH-HAE may be different in carriers of the three single nucleotide polymorphisms (SNP) of the *GR* gene because these SNPs have been associated with altered GC sensitivity. We hypothesized that they might have a role in mediating the effects of emotional stress on edema formation in patients with C1-INH-HAE, during attacks in the first place.

## Methods

### Patients

C1-INH-HAE group: All subjects had been diagnosed and receiving regular follow-up care at the Hungarian Angioedema Center. In each patient, we established the diagnosis of C1-INH-HAE according to standard clinical and laboratory criteria (positive family history, clinical symptoms of angioedema, low functional C1-INH level, low C4, normal C1q). During the scheduled visits, the time of occurrence, location, and severity of the edematous episodes were recorded along with the on demand therapy (e.g. C1-INH concentrate, icatibant) administered to relieve the attack. All these information was taken into account to modify long-term prophylaxis as necessary. Further, the concomitant medications taken on a regular basis and accompanying disorders were recorded, and the patients’ body height and weight were checked on these occasions.

The angioedema group comprised patients with angioedema, a negative family history, and normal C4, C1q, C1-INH antigen levels and functional activity.

Healthy controls: All had been referred for routine medical check-up, and volunteered for the study by giving informed consent. The healthy controls did not have any known disease (C1-INH deficiency was excluded by complement testing).

The study was approved by the institutional review board of Semmelweis University of Budapest. Informed consent was obtained from the subjects in accordance with the Declaration of Helsinki.

### Evaluation of the response to stress

The response of the subjects to stress was measured with Rahe’s Brief Stress and Coping Inventory [28]. This instrument is used to categorize the population tested into four subsets, according to subjectively experienced stress level and coping capabilities. The test was completed by 43 patients diagnosed with C1-INH-HAE (mean age: 38.00 years, SD: 16.87 years; 22 females and 21 males), by 18 patients showing angioedematosus symptoms without C1-INH deficiency (mean age: 48.00 years, SD: 19.56 years, 15 females and 3 males), and 13 healthy controls. Statistical analysis was performed with the Kruskal-Wallis test.

### Genotyping

We genotyped 139 patients diagnosed with C1-INH-HAE (mean age 38.9 years, range: 5–84 years, 76 females and 63 males). A Hungarian control population consisting of 160 healthy individuals was used for comparison as regards the prevalence of GR SNPs. Total genomic DNA was isolated from peripheral blood with a commercially available DNA isolation kit (QIAmp DNA Blood Mini Kit (Qiagen), according to the manufacturer’s instructions. The BclI and N363S polymorphisms were detected with allele-specific polymerase chain reaction (PCR), as described previously [14, 29].

The A3669G polymorphism was measured with a predesigned TaqMan SNP Assay (C_8951023_10) (Applied Biosystems, LifeTechnologies), by real-time PCR, according to the recommended protocol, on a 7500 Fast PCR System (Applied Biosystems, LifeTechnologies).

### Hormonal evaluation

Blood samples were collected from patients hospitalized (to the Semmelweis University, 3^rd^ Department of Internal Medicine) for an edematous attack. During the attack-free period, morning fasting blood samples were obtained from these patients between 8:00 and 11:00 AM at the Hungarian Angioedema Center of the 3rd Department of Semmelweis University. Blood cortisol levels were measured during edematous attacks in 36 C1-INH-HAE patients. The blood samples were obtained by antecubital venipuncture. The samples were stored refrigerated (at −70 °C) until the measurement of serum cortisol levels and of C1-INH activity. Total cortisol levels in the plasma were determined by electrochemiluminescence immunoassay (Elecsys Immunoanalyser System, Roche). The functional level of the C1-inhibitor was determined with an enzyme immunoassay kit (Quidel, USA).

### Statistical analysis

The allele frequencies of GR polymorphisms in C1-INH-HAE patients and in healthy controls were compared with Pearson’s *χ* ^2^ or Fisher’s exact test. The Hardy-Weinberg equilibrium was calculated for all polymorphisms. The associations between carrier status for polymorphisms and clinical or hormonal data were analyzed with ANOVA, and with the Kruskal-Wallis, or t-tests. We also performed statistical power analysis with a tool available online (https://www.dssresearch.com/KnowledgeCenter/toolkitcalculators/statisticalpowercalculators.aspx). Statistical power over 80%, and a p-value less than 0.05 were considered significant.

## Results

### The evaluation of response to stress

**Fig. 1:**
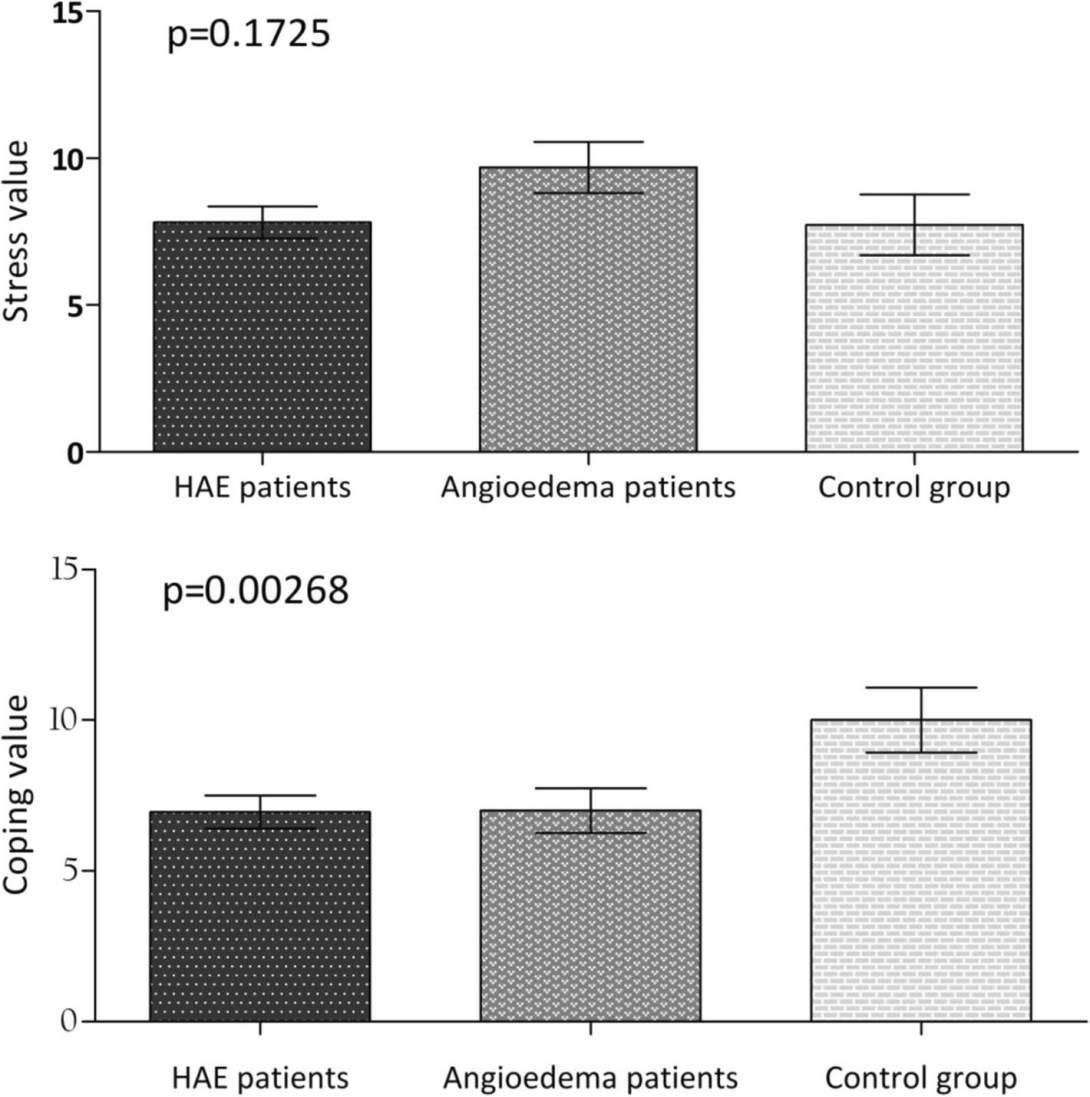
The scores achieved by the three subsets of our study population on Rahe’s Brief Stress & Coping Inventory. The scores achieved by the C1-INH-HAE patients, angioedema patients and healthy controls on Rahe’s Brief Stress & Coping Inventory

We did not find significant differences among the stress responses as measured with the Rahe’s Brief Stress and Coping Inventory tests in patients diagnosed with C1-INH-HAE, in angioedematous patients (without C1-INH deficiency), and healthy controls, using the Kruskal-Wallis one-way analysis of variance test (*p* = 0.1725). Reported coping capabilities differed significantly among the study populations (*p* = 0.0027). See Fig. 1.

### Hormonal evaluation

**Fig. 2:**
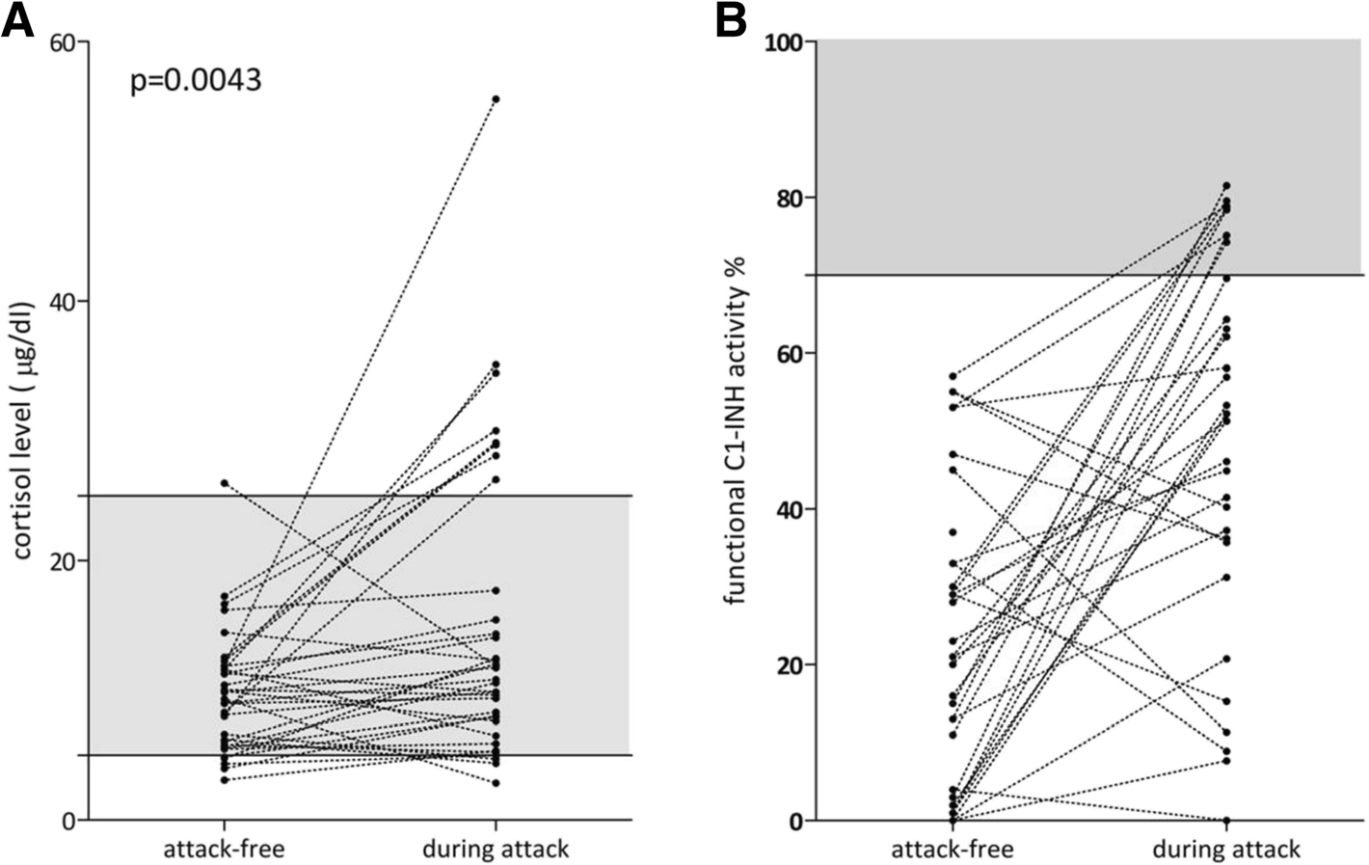
Serum total cortisol levels **a** and C1-INH activity **b** in the same patients between, or during edematous attacks. Serum total cortisol levels **a** and C1-INH **b** activity in blood samples obtained from the same patients between, or during edematous attacks. The reference range is marked by grey shading

Serum total cortisol levels were significantly (*p* = 0.004) different in samples obtained from the same patient during an edematous attack, or in an attack-free period (Wilcoxon matched pairs test) (Fig. 2). In particular, mean total cortisol level in the serum was 9.679 ug/dl (SD 4.68) during an attack-free period, and 14.89 ug/dl (SD 11.58) during an attack. Similarly, C1-INH activity was significantly (*p* < 0.0001) higher during attacks. While mean C1-INH activity was 22.88% (SD 18.98) in attack-free periods, it increased to 48.18% (SD 24.81) during attacks (Fig. 2). We did not detect a significant correlation between the changes of cortisol level and of C1-INH activity.

### GR polymorphisms

There was no difference between the two populations as regards the allele frequencies of the N363S, BclI and A3669G polymorphisms (Table 1). The A3669G homozygous carrier state was significantly lower in the C1-INH-HAE group compared to healthy controls (Statistical power: 71,4%).

**Table 1:** Minor allele frequency and carrier state for GR polymorphisms in C1-INH-HAE patients and in healthy controls

Polymorphism	C1-INH- HAE	Healthy control group	*p* value
	*N* = 139	*N* = 160	
N363S			
Minor allele frequency	0.05	0.031	0.78
Heterozygous carriers (+/−)	13 (9.3%)	10 (6.3%)	0.77
Homozygous carriers (+/+)	-	-	-
Non-carriers	126 (90.7%) (96,7%) (90.7%)	150 (93.7%)	0.77
BclI			
Minor allele frequency	0.36	0.35	0.87
Heterozygous carriers (+/−)	53 (38.1%)	82 (51.3%)	0.08
Homozygous carriers (+/+)	24 (17.3%)	16 (10%)	0.24
Non-carriers	62 (44.6%)	62 (38.7%)	0.72
A3669G			
Minor allele frequency	0.15	0.22	0.11
Heterozygous carriers (+/−)	39 (28.1%)	48 (30%)	0.99
Homozygous carriers (+/+)	2 (1.4%)	12 (7.5%)	**0.04**
Non-carriers	98 (70.5%)	100 (62.5%)	0.42

Minor allele frequency and carrier state for GR polymorphisms in C1-INH-HAE patients and in healthy controls. The p value was adjusted with Bonferroni correction. Statistically significant values are marked by bold typeset

### The association between A3669G polymorphism and cortisol levels in C1-INH-HAE patients

**Fig. 3:**
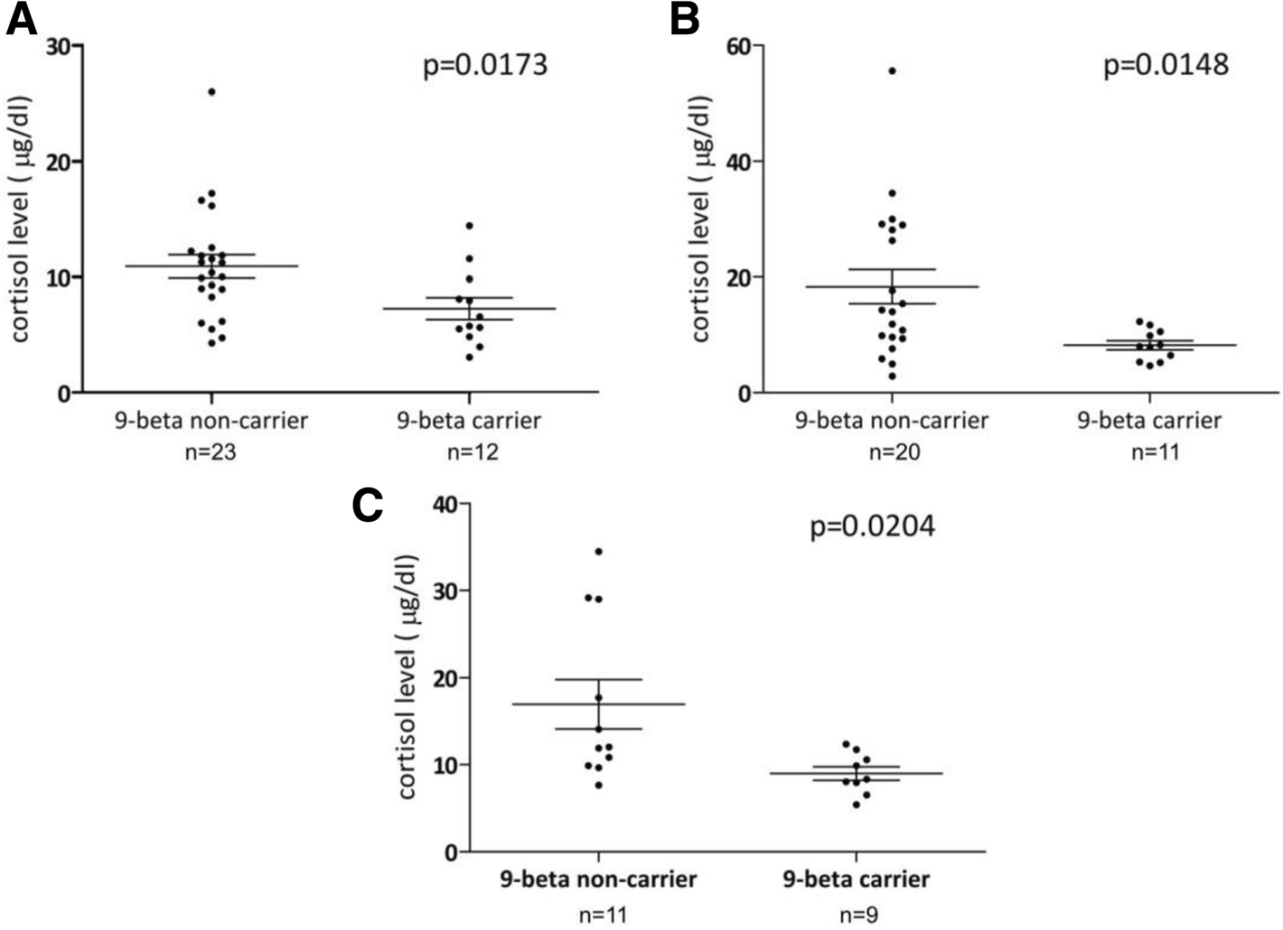
Cortisol levels in carriers and non-carriers of the A3669G polymorphism. Cortisol levels in carriers and non-carriers of the A3669G polymorphism. **a**: The entire patient population during attack-free period. **b**: Cortisol levels during attacks without upper airway edema. **c**: Cortisol levels during non-severe attacks

We grouped A3669G heterozygous and homozygous patients as A3669G carriers because of the low number of homozygous patients. Mean serum cortisol level was lower in carriers of the A3669G polymorphism compared to non-carriers (7.3 ± 3.3 vs. 10.9 ± 4.81, *p* = 0.0173; statistical power: 99.9%) (Fig. 3). Moreover the cortisol levels were lower during attack also in the carrier group; however, this difference did not reach significance (*p* = 0.0653).

In four patients, the attack was localized to the upper airway mucosa (pharynx and larynx) and caused obstruction. Such a symptom is a rather intense stressor and hence it may mask the impact of the polymorphism.

Therefore, we re-analyzed cortisol levels without the results of these patients. In A3669G carriers, lower basal cortisol levels remained significant (6.76 ± 3.14 vs. 10.96 ± 3.46, *p* = 0.013, statistical power: 92.9%). Meanwhile the difference between the steroid levels measured in the two groups during attacks had become significant (8.22 ± 2.64 vs. 18.34 ± 13.0394, p = 0.0148, statistical power 91.7%). Based on the previously mentioned hypothesis, patients who experienced severe attacks were also disregarded, but the difference between carrier and non-carrier groups nevertheless remained significant. Along similar considerations, we did not take into account the attacks rated severe by the patients themselves. Notwithstanding this, we found a significant difference between the two groups (8.94 ± 2.3 vs. 16.91 ± 9.4, p = 0.0204, statistical power 85.8%) (Fig. 3).

The change from baseline of the cortisol levels measured during edematous attacks was smaller in the A3669G carrier group compared with non-carriers, but this difference did not reach significance (1.00 ± 3.04 vs. 6.85 ± 14.40, *p* = 0.057).

We also found that A3669G carriers had significantly higher BMI values, whereas hypertension was more common in the group of BclI homozygous carriers, compared with non-carriers (Table 2). We did not find any association between the investigated polymorphisms and any other clinical variable (the initial onset of attacks, the frequency of edematous episodes, C1-INH consumption). There were no gender-specific associations between carrier status and hormonal levels.

**Table 2:** Clinical and metabolic parameters of C1-INH-HAE patients in relation to the investigated GR polymorphisms

	N363S		BclI			A3669G	
	Non-carriers *n* = 94	Carriers *n* = 12	Non-carriers *n* = 51	Heterozygous carriers *n* = 39	Homozygous carriers *n* = 16	Non-carriers *n* = 75	Carriers *n* = 31
	(54f + 40 m)	(5f + 7 m)	(31f + 20 m)	(20f + 19 m)	(8f + 8 m)	(40f + 35 m)	(19f + 12 m)
Age (mean ± SD)	39.88 ± 18.04	32.54 ± 11.88	35.02 ± 17.84	42.33 ± 15.99	43.79 ± 18.68	39.4 ± 18.2	39.2 ± 15.1
Onset of the initial attack (age) (mean ± SD)	12.23 ± 9.51	8.08 ± 5.68	11.71 ± 11.34	11.18 ± 6.58	13.3 ± 7.9	11.9 ± 8.8	11.2 ± 10.2
Number of attacks /year (mean ± SD)	8.4 ± 11.2	11.9 ± 13.3	10.2 ± 13.6	7.4 ± 9.9	7.5 ± 6.3	8.4 ± 12.4	10 ± 8.9
Average C1-INH consumption (amp/year) (mean ± SD)	1.6 ± 3.5	2.4 ± 3.9	1.8 ± 4.0	1.6 ± 3.1	1.6 ± 3.2	2.2 ± 3.5	1.5 ± 3.6
BMI (kg/m^2^) (mean ± SD)	24.99 ± 5.55	27.29 ± 3.91	24.17 ± 5.56	25.99 ± 5.21	26.87 ± 5.11	**24.2 ± 5.03°**	**28.4 ± 4.8°**
Prevalence of hypertension (%)	18.1	8.33	7.80*	17.95	37.5*	17.3	16.1
Prevalence of diabetes (%)	5.3	0%	3.9	5.13	6.2	5.3	3.2

Clinical and metabolic parameters of C1-INH-HAE patients in relation to the investigated GR polymorphisms. Results are mean ± SD. Statistically significant values are marked by bold typeset. °*p* < 0.0001 statistical power 98.2%, *p = 0.0126, statistical power 84.6%

*Abbreviations*: *f* female, *m* male.

## Discussion

In this study, we showed that stress response is intact in patients with C1-INH-HAE, although the reported coping capabilities differed significantly among the subsets of the study population. The lifelong management of any chronic and/or life-threatening disease requires considerable mental strength [30]. This might have contributed to the C1-INH-HAE-patients’ propensity for depression. The latter we have investigated in a previous study, the findings of which are in agreement with those published by *Fouche et al.* [31].

During stress, activation of the HPA axis results in the elevation of stress hormone levels: the serum concentrations of cortisol and of catecholamines reflect the activation of HPA axis. In our patient population, basal cortisol level was lower in C1-INH-HAE patients carrying the A3669G polymorphism. This SNP increases the stability of the splicing variant GRß [24], which inhibits the function of GRα. Our results are consistent with those reported by van *Schoor et al.*, who found reduced serum fasting cortisol levels in female carriers of the A3669G polymorphism, compared with homozygous carriers of the wild type [17]. In stressful situations, the elevation of the cortisol levels of C1-INH-HAE patients during an edematous attack might result from the activation of HPA axis. This offers a possible, alternative explanation for the increase of white blood cell count during attacks described previously by our study group [32], which previously has been attributed to haemoconcentration. Remarkably, during non-severe attacks, carriers of the A3669G polymorphism had lower cortisol levels, and exhibited a smaller elevation of serum cortisol level than non-carriers. This suggests blunted responsiveness of the HPA axis – in agreement with the findings by *Kumsta et al*. These authors reported higher awakening ACTH and salivary cortisol levels after dexamethasone administration in male A3669G carriers [33]. Remarkably, they also found that healthy male carriers of the A3669G minor allele showed the highest ACTH and cortisol levels in response to social stress; however this observation was not confirmed by a subsequent study in adolescents [33, 34]. These somewhat controversial results on the association between polymorphisms and cortisol levels under stress may be related to the differences in the study populations and stressors. Nevertheless, there is strong evidence that polymorphisms in the *GR* gene gene thorough the negative feed-back effect of cortisol on the HPA axis may modify the responsiveness of the HPA, along with individual stress responses [19]. Together, these data confirm that the A3669G carrier state is associated with relative glucocorticoid resistance during activation of the HPA axis. In C1-INH-HAE patients, edematous attacks are a chronic source of stress, permanently elevated glucocorticoid levels due to the chronic activation of the HPA axis may lead to the development stress related disorders, eg. dysfunction of the immune system, hypertension, diabetes and adverse cardiovascular events. Hypothetically alterations of HPA axis responsivity may influence these unfavourable outcomes of chronic stress, however the impact of GR polymorphisms on stress response needs further examinations, including the measurement of ACTH and prospective follow up of patients.

Stress responsiveness, and activation of the HPA axis are known to differ between the sexes [35]. Furthermore, *Kumsta et al.* found gender-specific differences in the modulation of the responsiveness of the HPA axis by GR polymorphisms [33]. However, we could not observe sex-specific associations in our study.

We found that the allelic frequencies of the investigated three polymorphisms in the *GR* gene (BclI, N363S, A3669G) did not differ significantly between C1-INH-HAE patients and healthy controls. Although the A3669G homozygous carrier state was significantly lower in C1-INH-HAE patients, the low statistical power rather indicate this finding a bias.

We could not detect any relationship between the investigated GR polymorphisms and the severity of edematous attacks (as regards attack frequency, and C1-INH consumption) in C1-INH-HAE patients. Furthermore, the during-attack elevation of C1-INH functional levels did not exhibit any correlation with cortisol levels. These data suggest that glucocorticoids are not involved in the mechanism of edema formation due to C1-INH deficiency.

Glucocorticoids play an important role in the regulation of metabolism. Polymorphisms in the *GR* gene have been previously linked with various clinical parameters [11]. In our C1-INH-HAE patients, the prevalence of hypertension was higher in carriers of the polymorphic BclI allele. BclI polymorphism has been implied with an increased response to glucocorticoids. Our results are in accord with earlier observations regarding the unfavorable effect of BclI polymorphisms on blood pressure in different patient populations [36–38]. Interestingly, carriers of the A3669G allele had increased BMI. This is rather intriguing, as lower serum cortisol levels are expected to protect the carriers against weight gain. This finding suggests a poor correlation among blood cortisol levels and metabolic parameters.

## Conclusions

In summary, the examined polymorphisms of the *GR* gene are most likely not involved in the pathomechanism of C1-INH HAE. Minor allele carriers of the A3669G polymorphism have lower cortisol levels both in attack free periods, and during attacks. Possibly, this reflects a relative resistance against glucocorticoids on the level of the HPA axis. In contrast with this observation, we could not find any association between carrier state and disease severity in HAE patients. Further hormonal evaluations are necessary to clarify the impact of *GR* polymorphisms on the responsiveness of the HPA axis in C1-INH-HAE patients.

## Abbreviations

BMI: Body mass index
C1-INH: C1-inhibitor
C1-INH-HAE: Hereditary angioedema with C1-inhibitor deficiency
CAH: Congenital adrenal hyperplasia
GC: Glucocorticoid
GCs: Glucocorticoids
GR: Glucocorticoid receptor
GRß: Glucocorticoid receptor beta
GRα: Glucocorticoid receptor alpha
HAE: Hereditary angioedema
HPA: Hypothalamic–pituitary–adrenal
PCR: Polymerase chain reaction
RFLP: Restriction fragment length polymorphism
SNP: Single nucleotide polymorphisms.
